# Olfactory swab sampling optimization for α-synuclein aggregate detection in patients with Parkinson’s disease

**DOI:** 10.1186/s40035-022-00311-3

**Published:** 2022-07-28

**Authors:** Matilde Bongianni, Mauro Catalan, Daniela Perra, Elena Fontana, Francesco Janes, Claudio Bertolotti, Luca Sacchetto, Stefano Capaldi, Matteo Tagliapietra, Paola Polverino, Valentina Tommasini, Giulia Bellavita, Elham Ataie Kachoie, Roberto Baruca, Andrea Bernardini, Mariarosaria Valente, Michele Fiorini, Erika Bronzato, Stefano Tamburin, Laura Bertolasi, Lorenzo Brozzetti, Maria Paola Cecchini, Gianluigi Gigli, Salvatore Monaco, Paolo Manganotti, Gianluigi Zanusso

**Affiliations:** 1 Department of Neurosciences, Biomedicine, and Movement Sciences, Policlinico G. B. Rossi, University of Verona, 37134 Verona, Italy; 2 Neurology Unit, Department of Medicine, Surgery and Health Sciences, Ospedale Cattinara, University of Trieste, 34128 Trieste, Italy; 3 Neurology Unit, University of Udine Academic Hospital, 33100 Udine, Italy; 4 Department of Surgical Sciences, Dentistry, Gynecology and Pediatrics, University of Verona, 37134 Verona, Italy; 5 Biocrystallography Laboratory, Department of Biotechnology, University of Verona, 37134 Verona, Italy; 6 Otolaryngology Unit, Department of Medicine, Surgery and Health Sciences, Ospedale Cattinara, University of Trieste, 34128 Trieste, Italy

**Keywords:** Parkinson disease, Alpha-synuclein, Real-time quaking-induced conversion assay, Olfactory mucosa, Cerebrospinal fluid

## Abstract

**Background:**

In patients with Parkinson’s disease (PD), real-time quaking-induced conversion (RT-QuIC) detection of pathological α-synuclein (α-syn) in olfactory mucosa (OM) is not as accurate as in other α-synucleinopathies. It is unknown whether these variable results might be related to a different distribution of pathological α-syn in OM. Thus, we investigated whether nasal swab (NS) performed in areas with a different coverage by olfactory neuroepithelium, such as *agger nasi* (AN) and middle turbinate (MT), might affect the detection of pathological α-syn.

**Methods:**

NS was performed in 66 patients with PD and 29 non-PD between September 2018 and April 2021. In 43 patients, cerebrospinal fluid (CSF) was also obtained and all samples were analyzed by RT-QuIC for α-syn.

**Results:**

In the first round, 72 OM samples were collected by NS, from AN (NS^AN^) or from MT (NS^MT^), and 35 resulted positive for α-syn RT-QuIC, including 27/32 (84%) from AN, 5/11 (45%) from MT, and 3/29 (10%) belonging to the non-PD patients. Furthermore, 23 additional PD patients underwent NS at both AN and MT, and RT-QuIC revealed α-syn positive in 18/23 (78%) NS^AN^ samples and in 10/23 (44%) NS^MT^ samples. Immunocytochemistry of NS preparations showed a higher representation of olfactory neural cells in NS^AN^ compared to NS^MT^. We also observed α-syn and phospho-α-syn deposits in NS from PD patients but not in controls. Finally, RT-QuIC was positive in 22/24 CSF samples from PD patients (92%) and in 1/19 non-PD.

**Conclusion:**

In PD patients, RT-QuIC sensitivity is significantly increased (from 45% to 84%) when NS is performed at AN, indicating that α-syn aggregates are preferentially detected in olfactory areas with higher concentration of olfactory neurons. Although RT-QuIC analysis of CSF showed a higher diagnostic accuracy compared to NS, due to the non-invasiveness, NS might be considered as an ancillary procedure for PD diagnosis.

**Supplementary Information:**

The online version contains supplementary material available at 10.1186/s40035-022-00311-3.

## Introduction

Parkinson’s disease (PD) is the most common form of parkinsonism, clinically characterized by motor symptoms (e.g., bradykinesia, tremor, rigidity, and postural instability) and non-motor symptoms (e.g., cognitive decline, depression, overactive bladder, and constipation) [1, 2]. Several years before the clinical onset, PD patients might complain of hyposmia and rapid eye movement sleep behavior disorder (RBD) [1, 2]. Around 80% of patients with PD eventually develop dementia later in the disease course [3]. Neuroimaging investigations such as magnetic resonance imaging (MRI) and striatal dopamine-transporter imaging are supportive of clinical diagnosis.

The definitive diagnosis of PD is only achieved at autopsy. The detection of intracellular deposits of α-synuclein (α-syn) aggregates forming Lewy bodies is the neuropathological hallmark of PD [4]. Therefore, the detection of α-syn aggregates in biofluids or peripheral tissues of patients with PD would be essential in clinical practice for early diagnosis and prognostic assessment. In this context, a novel assay called real-time quaking induced conversion (RT-QuIC) has shown capability to amplify trace amounts of α-syn aggregates in cerebrospinal fluid (CSF) from patients with PD, with variable diagnostic sensitivity (~ 80%–90%) and specificity (~ 90%–100%) [5–9]. Using RT-QuIC, α-syn aggregates propagate with a prion-like replication mechanism by inducing conversion of recombinant α-syn to the misfolded form. Then, the converted α-syn initiates amyloid fibril formation which, in turn, enhances the fluorescence of Thioflavin T (ThT).

Parallel studies have been conducted to identify easily accessible peripheral tissues such as olfactory mucosa (OM) and skin of patients with PD, multiple system atrophy (MSA) and dementia with Lewy bodies (DLB) with reliable diagnostic robustness [10–14]. As opposed to CSF, in which α-syn aggregates are dissolved homogeneously, in peripheral tissues a regional distribution of α-syn aggregates has been assessed. In this context, seminal post-mortem studies on PD have identified distinct areas of peripheral tissues (i.e., skin, submandibular glands or gastroenteric mucosa) with the highest concentration of phospho-α-syn. These findings were subsequently conveyed in patients for obtaining successful tissue sampling [15, 16]. In skin, a map of α-syn aggregate distribution shows a higher distribution in the cervical region, thigh, and leg [17]. Skin biopsies from patients with PD display phospho-α-syn deposits in dermal nerves and RT-QuIC testing showed sensitivity and specificity of 77%–96% and 95.1%–100%, respectively [13, 14, 17, 18]. Submandibular glands also show variable phospho-α-syn deposits positive to RT-QuIC, but these studies are on autoptic samples and clinical studies are still pending [19].

The OM has been targeted for RT-QuIC analysis of α-syn in patients with PD, MSA and DLB, with a relevant diagnostic accuracy in MSA (82%) and DLB (86.4%), but not in PD patients (range from 44% to 48%) [10–12].

**Fig. 1:**
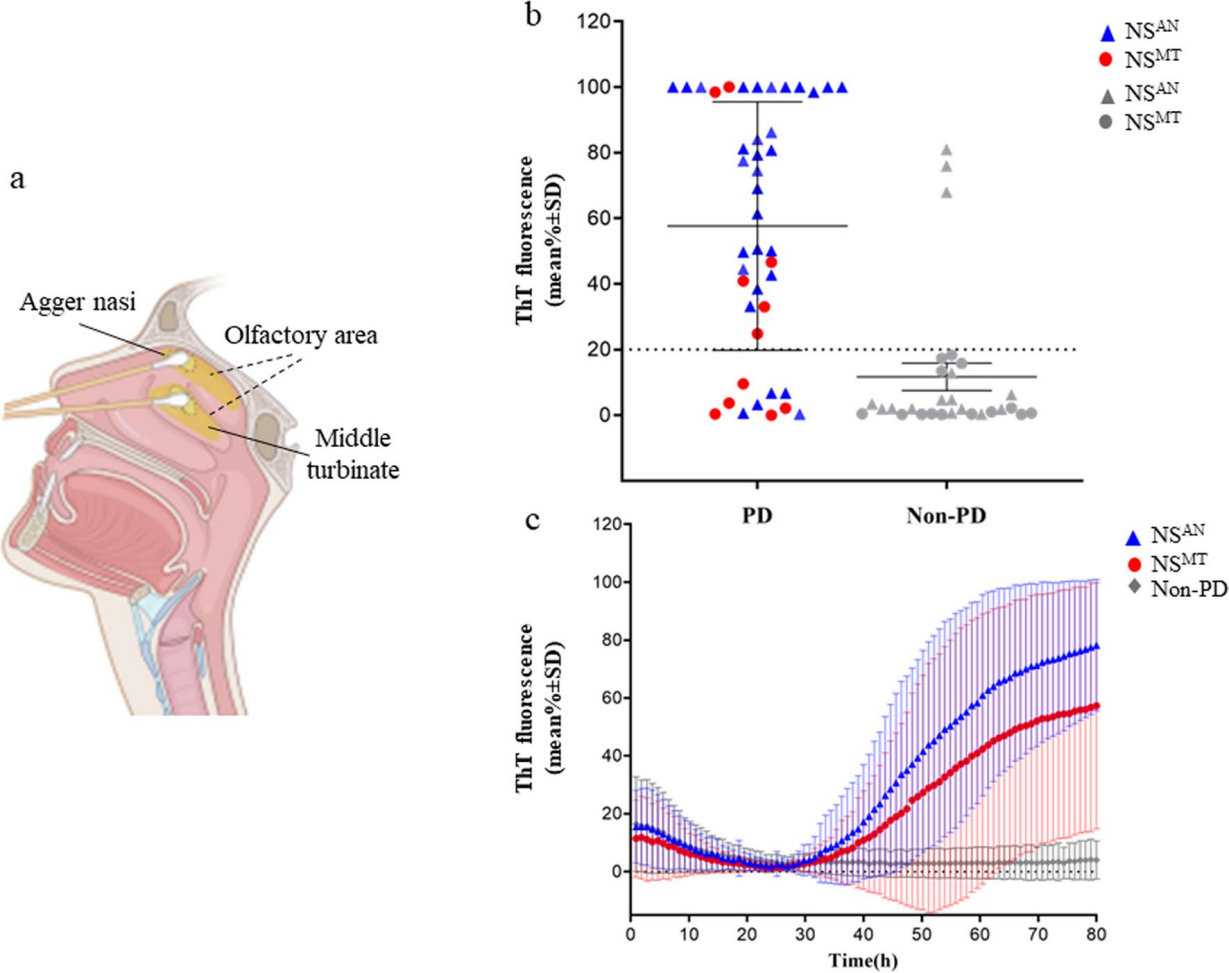
RT-QuIC detection of α-syn seeding activity in the nasal swabs (NSs) of patients with PD and non-PD. **a** Illustration of olfactory mucosa (OM) swab sampling from *agger nasi* (AN) (NS^AN^) and middle turbinate (MT) (NS^MT^) (created with BioRender.com). **b** Average thioflavin T (ThT) fluorescence from four replicate readings obtained in NS for each subject with PD (*n* = 43) and non-PD (*n* = 29) at 80 h. NS^AN^ are shown as blue triangles (*n* = 46) while red dots denote NS^MT^ (*n* = 26). **c** Traces represent the relative average percentage of ThT fluorescence readings from four replicate reactions (normalized as described in the Methods section) from PD and non-PD samples. The means (thick lines) with standard deviations (thin lines) are shown as a function of RT-QuIC reaction time

Given the previous evidence of the low sensitivity of RT-QuIC test in OM of PD patients, we explored the performance of RT-QuIC in samples collected from two different regions of OM, the AN, a small ridge located in the top of the nasal vault, and the MT (Fig. 1a). These areas were chosen for their different coverage by olfactory neuroepithelium, known to be more represented at the level of AN [20, 21]. In addition, in smears of NS we aimed to characterize the cell population and to assess the level of α-syn and phospho-α-syn expression. Finally, in a restricted number of patients the diagnostic accuracy of RT-QuIC was also tested in the CSF as compared with the OM.

## Methods

### Ethics statement

The study was conducted according to the revised Declaration of Helsinki and Good Clinical Practice guidelines. Informed consent was given by study participants or the next-of-kin. OM or CSF sample collection was performed under protocols approved by the Ethical Committee of each unit, University of Trieste (UNITS) (CEUR-2020-Sper-013, February 11th, 2020), University of Udine (UNIUD) (UD: 26499) and University of Verona (UNIVR) (Prot. n. 28917 June 15th, 2012). Written informed consent was obtained from patients or legal representatives.

### Patients and clinical assessment

Patients were recruited at three neurology units including UNITS, UNIUD and UNIVR. Sixty-six patients received a clinical diagnosis of probable PD based on the international diagnostic criteria [2, 3]. Additional 29 patients with other clinical diagnoses, based on established diagnostic criteria, such as Alzheimer’s disease (AD) (*n* = 13), progressive supranuclear palsy (PSP) (*n* = 6), Creutzfeldt-Jakob disease (CJD) (*n* = 7) and essential tremor (*n* = 3), were recruited as non-PD patients. Based on the clinical history and neurological evaluation performed by neurology specialists in neurodegenerative disorders, appropriate diagnostic investigations were performed, including MRI, FDG PET, ^123^I-Ioflupane SPECT and CSF measurement of biomarkers of dementia. Next, for each patient a final diagnosis was made by applying the latest recommended criteria for PD, AD, PSP, CJD and essential tremor (Table 1) [22–25]. RT-QuIC analysis of OM and CSF samples was carried out at UNIVR by clinical diagnosis investigators who were blinded to the patients’ diagnosis. All patients included in this study underwent NS and 43 of them further underwent CSF collection. For immunocytochemical study, NS samples from 12 healthy volunteers (6 males and 6 females, mean age 56 ± 5 years) were also included.

**Table 1:** Demographics, clinical characteristics and RT-QuIC results of PD patients who underwent NS and CSF sampling

	Patients underwent NS at the (*n* = 46)		Patients underwent NS at the MT (*n* = 26)		*P*-value (effect size)	
	PD (*n* = 32)	Non-PD (*n* = 14)	PD (*n* = 11)	Non-PD (*n* = 15)	AN *vs* MT in PD	AN vs MT in non-PD
Age at diagnosis, years	67 (60–70)	73 (70–76)	65 (53–70)	68 (64–75)	0.3 (*R*^2^ = .02)	0.2 (*R*^2^ = 0.07)
Sex Female/Male	12/20	4/10	3/8	5/10	0.7 (φ_c_ = 0.09)	0.9 (φ_c_ = 0.05)
Age at onset, years	66 (59–69)	70 (61–74)	64 (51–69)	68 (63–71)	0.4 (*R*^2^ = 0.02)	0.4 (*R*^2^ = 0.02)
Disease duration (from diagnosis), years	2 (1–3)	ND	2 (1–2)	ND	0.9 (*R*^2^ = 0.00)	–
Interval between clinical diagnosis and NS, months	1 (1–8)	12 (12–24)	12 (2–12)	12 (6–24)	0.009 (*R*^2^ = 0.17)*	0.9 (*R*^2^ = 0.00)
Interval between lumbar puncture and NS, months	1 (1–3) (*n* = 23)	12 (1–18) (*n* = 8)	2 (2–2) (*n* = 1)	2 (2–12) (*n* = 11)	0.6 (*R*^2^ = 0.04)	0.7 (*R*^2^ = 0.01)
MDS-UPDRS III score	18 (11–33)	5 (2–17)	20 (12–31)	ND	0.9 (*R*^2^ = 0.02)	^* –*^
MoCA score	27 (24–29)	21 (19–24)	25 (24–30)	ND	0.5 (*R*^2^ = 0.01)	^* –*^
Hyposmia *n* (%)	17/32 (53)	ND	5/11 (46)	ND	0.7 (φ_c_ = 0.07)	^* –*^
RT-QuIC-positive OM (%)	27 (84)*	2 (14)	5 (45)*	1 (7)	0.018 (φ_c_ = 0.39)*	0.6 (φ_c_ = 0.12)
RT-QuIC-positive CSF (%)	21/23 (91)	1/8 (12)	1/1	0/11	0.76	0.4

Data are shown as median (IQR) or *n* (%)

AN, agger nasi; MT, middle turbinate; ND, not determined

*Significant differences in group comparisons; –, not tested due to extreme or low counts

### OM sample collection

NS was performed by otolaryngologists in each unit, independently. A step-by-step tutorial video of NS procedure is available at https://www.youtube.com/watch?v=wYb9W3u6uMY. At UNITS and UNIUD, NS procedure was performed at the level of AN and the patients were assigned in the NS^AN^ group. At UNIVR, OM samples were collected through the MT and this group of patients was named NS^MT^ (Fig. 1a). Two to four OM samples were collected from both sides in each subject, depending on individual’s tolerability. The procedure was well tolerated by all subjects without complications and we did not observe individual reluctance to NS, which is a well-known test for COVID-19. Following the NS procedure, swabs were placed in polypropylene tubes containing saline or fixative (Diacyte, Diapath, Martinengo, Italy) solutions, sealed and sent to UNIVR. Upon arrival, tubes were vortexed and centrifuged at 1700 rpm for 15 min. The OM pellets in saline were frozen and kept at −80 °C for RT-QuIC analyses. The fixed cells were counted with Countess™ II FL Automated Cell Counters (Invitrogen) and diluted if necessary. Cell suspension was cyto-centrifuged onto slides using a cytospin (CYTOSPIN IV, AHSI, Italy) and slides were kept frozen until immunostaining [26].

### Immunocytochemical analysis

NS samples collected from AN and MT of 18 patients with PD were processed for immunocytochemistry. Slides were incubated overnight at 4 °C with primary anti-β-tubulin III (1:400; T2200 Sigma), anti-α-syn (1:400; BioLegend) and anti-phospho-α-syn (1:1000; Invitrogen, Waltham, MA) antibodies. On the next day, the slides were washed and incubated for 1 h at room temperature with Alexa Fluor-conjugated secondary antibodies (1:1000, Life Technologies, Waltham, MA). After washing, the slides were incubated with DAPI (1:2000) for 5 min and mounted with DABCO (Sigma-Aldrich, Milan, Italy). Images were acquired using the Axiolab fluorescence microscope (Zeiss, Oberkochen, Germany).

### Expression and purification of recombinant human α-synuclein

Recombinant α-syn was expressed and purified from the periplasmic fraction as reported [27]. Briefly, wild-type human α-syn cDNA was cloned in the pET-28a plasmid (Novagen, Milan, Italy) and transformed into Escherichia coli BL21(DE3). Cell cultures (1 L) were grown at 37 °C to an OD600 of 0.3–0.4 and the expression was induced with 0.1 mM isopropyl b-*D*-1-thiogalactopyranoside for 5 h. The cells were collected by centrifugation, resuspended in 100 ml of osmotic shock buffer (30 mM Tris-HCl pH 7.2, 40% sucrose, and 2 mM EDTA) and incubated for 10 min at room temperature. The pellet was centrifuged at 12,000 rpm, resuspended in 90 ml of cold water added with 37.5 µl of saturated MgCl_2_ solution and centrifuged again after 5-min incubation on ice. The supernatant containing periplasm proteins was boiled for 15 min and cleared by centrifugation. The soluble fraction, enriched in α-syn, was subjected to ammonium sulfate precipitation followed by extensive dialysis against 20 mM Tris–HCl, pH 8.0. Alpha-syn was further purified by anion exchange chromatography: the protein was loaded on a Q-Sepharose column (GE Healthcare, Milan, Italy) equilibrated with the same buffer and eluted with a 0–500 mM linear gradient of NaCl. The purity of α-syn was checked by SDS-PAGE. The protein was then dialyzed against 10 mM sodium phosphate buffer pH 7.4 and stored at -80 °C until use.

### Alpha-synuclein RT-QuIC in OM swabs

OM samples were thawed and a disposable inoculating loop (Fisherbrand, Milan, Italy) was dipped into the pellet to transfer ∼2 μl of the pellet into a tube containing 120 μl phosphate-buffered saline (PBS). The latter tube was sonicated at 120 W (Digital ultrasonic bath Mod.DU-32, Argo Lab, Carpi, Italy) for at least 1 min until the pellet was dispersed. For each test, 2 μl of the diluted OM sample was plated in 98 µl of reaction buffer composed of 100 mmol/l phosphate buffer (pH 8.2), 10 μmol/l ThT, and 0.05 mg/ml human recombinant full-length (1–140 aa) α-syn and 37 ± 3 mg of 0.5 mm glass beads (Sigma). The plate was sealed with a plate sealer film (Nalgene Nunc International, Rochester, NY) and then incubated at 30 °C in a BMG FLUOstar® Omega plate reader with cycles of 1-min shaking (200 rpm double orbital) and 14-min rest. ThT fluorescence measurements (450 ± 10 nm excitation and 480 ± 10 nm emission; bottom read) were taken every 45 min. Four replicate reactions were tested for each sample.

### RT-QuIC analysis in NS and CSF samples

Alpha-syn RT-QuIC test has been previously optimized in OM and CSF samples from patients with PD, DLB and RBD, as described [11, 12, 27]. Four replicate reactions were tested for each sample. A sample was considered positive when at least two of four replicate wells crossed the calculated threshold (20% maximum ThT fluorescence). In detail, for each set of replicate reactions, the mean baseline relative fluorescence units (rfu) value was calculated over a 10-h period spanning the lowest part of a plot of the mean rfu of all replicates versus reaction time. This value was then subtracted from the mean rfu values at each time point to give the baseline‐adjusted mean rfu values. The latters were then normalized as percentages of the baseline‐adjusted maximal fluorescence rfu values as follows: baseline-adjusted rfu values/baseline-adjusted maximum rfu value × 100% [11, 12]. These normalized values were plotted versus reaction time. ThT fluorescence positivity threshold was calculated as the average fluorescence for all samples between 15 and 17 h of incubation plus three standard deviations (SD). Cut-off time was assessed at 80 h for both NS and CSF samples, based on the results from definite cases, in order to obtain the best specificity and sensitivity. Absence of RT-QuIC seeding reaction was also determined in OM samples from PD patients without substrate and substrate without OM samples (Additional file 1: Fig. S1).

## Statistical analysis

RT-QuIC relative fluorescence responses were analysed and plotted using the software Graphpad Prism 8.3. We compared the mean relative ThT fluorescence and the lag phase responses in NS and CSF samples by either two-tailed unpaired *t*-test or by following ascertainment of normal distribution of data by Shapiro–Wilk normality test. Numerical variables were assessed using histograms, Q-Q plots and Kolmogorov–Smirnov’s test for normality. First, we tested the proportions of positive OM and CSF samples, demographic, clinical and instrumental data from the two centres in the original cohort for homogeneity using Fisher’s exact test and Kruskal–Wallis H test for numerical variables. Cramer’s V (φ_c_) and Eta-squared (${\eta }_{H}^{2}$) were used to estimate the effect size. We then tested the same variables from the supplementary cohort and the original cohort for homogeneity using Fisher’s exact test for binomial variables and Mann–Whitney U-test for numerical variables. Cramer’s V (φc) and R^2^ were used to estimate the effect size. Finally, agreement between the anterior and posterior sampling methods in the supplementary cohort was tested for different results by McNemar test, using Cohen’s kappa (*k*) to estimate the effect size. *P* values < 0.05 were considered statistically significant.

## Results

### NS sampling and RT-QuIC assay in patients with PD and other diagnoses

At the first round, 72 patients with PD and other neurodegenerative disorders underwent NS (Table 1). In total, 72 OM samples were obtained, including 46 AN samples from 32 PD patients and 14 non-PD, and 26 MT samples 11 PD patients and 15 non-PD.

Thirty-five samples were positive for α-syn in RT-QuIC. In particular, 32 samples were from patients having a clinical diagnosis of probable PD while 3 were from non-PD patients, including one patient with a clinical diagnosis of AD, one with PSP and one with CJD (Fig. 1b). As such, α-syn RT-QuIC was positive in OM of 32/43 (74%) patients with PD and 3/29 (10%) patients with non-PD. The proportion of positive samples from PD patients was significantly higher in NS^AN^ (27/32, 84%) compared to NS^MT^ (5/11, 45%) (*P* = 0.018, φ_c_ = 0.39) (Table 1). Interestingly, among the 32 PD patients positive for RT-QuIC α-syn, 17 complained of hyposmia (53%), but 4 out of 5 patients with AN sampling who were OM negative did not complain of olfactory dysfunction.

In addition, the average α-syn seeding reactivity of NS^AN^ samples was higher than that of NS^MT^ samples (the maximum ThT fluorescence at the endpoint of 80 h was 80% for NS^AN^ compared to 57% for NS^MT^; *P* < 0.05), and the average reaction time required to exceed the designated positivity threshold was 41 h for NS^AN^ and 47 h for NS^MT^ (see also Materials and Methods) (Fig. 1c).

### Evaluation of RT-QuIC sensitivity in OM samples obtained at the level of AN and through the MT

To confirm that the RT-QuIC sensitivity would be higher when OM samples were obtained at the AN, 23 additional patients with PD were recruited. The demographic and clinical characteristics of the PD patients for OM swab sampling did not differ from the first group of patients (Table 2) and each patient underwent OM swab sampling either at the AN or through the MT.

**Table 2:** Comparative analysis of demographic and clinical characteristics between PD patients tested at the first and the second rounds

	PD patients of the first round (*n* = 43)	PD patients of the second round (*n* = 23)	*P* value
Age at diagnosis, years	66 (59–70)	70 (61–75)	0.14
Sex female/male	15/28	7/16	0.79
Age at onset, years	65 (57–69)	68 (60–71)	0.24
Interval between clinical diagnosis and NS, months	2 (1–12)	2 (1–12)	0.99
MDS-UPDRS III score	20 (12–32)	18 (9–33)	0.95
MoCA score	26 (24–29)	24 (24–28)	0.11
RT-QuIC-positive NS^AN^ (%)	27/32 (84)	18/23 (78)	–
RT-QuIC-positive NS^MT^ (%)	5/11 (45)	10/23 (43)	–

Data are shown as median (IQR) or *n* (%)

**Fig. 2:**
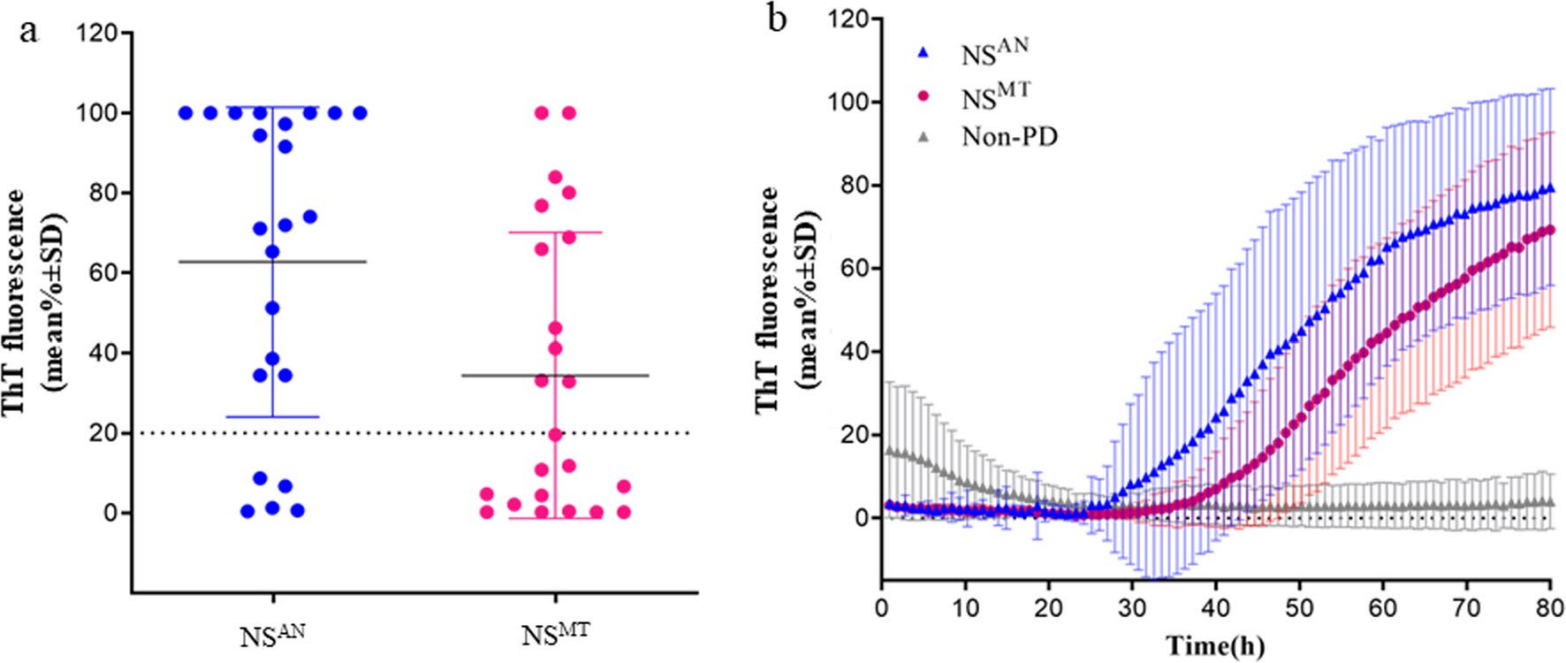
RT-QuIC detection of α-synuclein seeding activity in the nasal swabs (NS) of PD patients at *agger nasi* (AN) and middle turbinate (MT). **a** The final average relative ThT fluorescence from four replicate readings obtained from NS of each individual case (*n* = 23) at the level of AN and through the MT at 80 h. Bars show the average ± SD for all the cases in each group. The dashed line shows the fluorescence threshold for a positive result. **b** Traces represent the relative average percentage of ThT fluorescence readings of positive samples from AN (blue trace) and the MT (magenta trace), and non-PD (grey trace) as negative controls. The means (thick lines) with SDs (thin lines) of those averages are shown as a function of RT-QuIC reaction time

Different proportions of RT-QuIC-positive samples were observed between NS^AN^ and NS^MT^. In particular, NS^AN^ resulted RT-QuIC positive in 18 out of 23 (78%), while NS^MT^ in 10 out of 23 (44%) (Fig. 2a) (*κ* = 0.19, 95% CI 0.00 to 0.49, *P* = 0.021). In particular, four patients were identified to be negative for α-syn by both NS^AN^ and NS^MT^ RT-QuIC; nine patients were identified to be positive for α-syn by NS^AN^ RT-QuIC but negative by NS^MT^ RT-QuIC; and one patient was negative in the NS^AN^ RT-QuIC but positive in the NS^MT^ RT-QuIC. In addition, the average seeding reactivity of the positive samples was statistically higher in NS^AN^ than in NS^MT^, similar as that in the first round of testing (79% and 69% maximum ThT fluorescence at 80 h) (*P* < 0.05) (Fig. 2b).

These results suggest similar RT-QuIC sensitivity between the first and the second rounds, in both NS^AN^ (84% in the first *vs* 78% in the second round) and NS^MT^ (45% *vs* 43%). Moreover, the results of average α-syn seeding activity were also similar between the first and the second rounds in both NS^AN^ and NS^MT^, confirming the higher seeding reactivity of α-syn aggregates in NS^AN^.

### Immunocytochemical analysis of NS^AN^ and NS^MT^

**Fig. 3:**
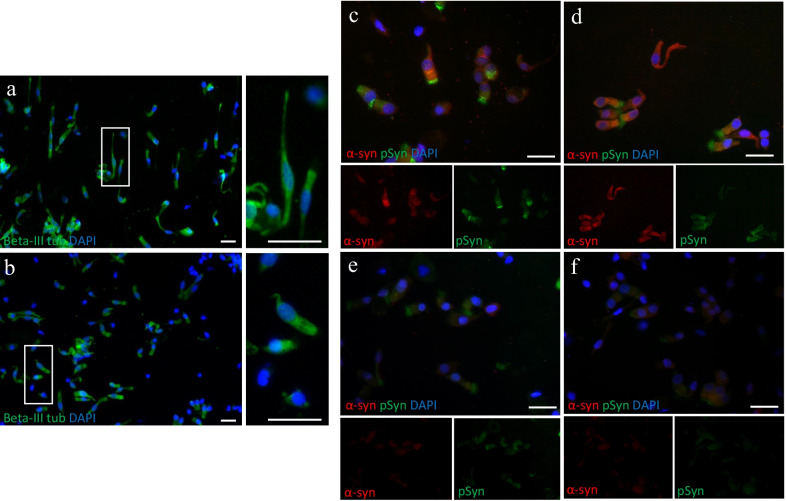
Immunocytochemical analyses of nasal swabs (NS) of controls and PD patients. **a**, **b** OM cell preparations from NS of AN (**a**) and MT (**b**) of a PD patient and immunostained with anti-β-tubulin III antibody (green) (scale bars 25 µm, magnification 20×). Thin and elongated β-III tubulin-positive cells with a neuronal-shape morphology are dominant in AN (inset, scale bar 25 µm) compared to MT (inset, scale bar 25 µm). **c**, **d** Immunostaining with anti-α-syn (red) and anti-phospho-α-syn (green) antibodies of NS from the PD patient showed α-syn and phospho-α-syn deposits in neuronal-shaped and ciliated cells. In contrast, NS from a non-PD patient (**e**) and normal control (**f**) showed a weak positivity (scale bar 25 µm, magnification 40×). Nuclei were stained with DAPI (blue)

We next aimed to demonstrate that RT-QuIC results obtained from NS^AN^ and NS^MT^ correlated with a different representation of olfactory neuroepithelium. OM samples from 18 PD patients were immunostained with anti-β-tubulin III antibody, a phenotypic marker of olfactory neuron and its precursors, and a higher number of β-III tubulin-positive cells was observed in NS^AN^ compared to NS^MT^ (Fig. 3a *vs* b). Furthermore, the OM preparations were immunostained for α-syn and phospho-α-syn. In NS from 18 PD patients, a diffuse α-syn positivity was observed in olfactory neurons and supporting cells. Sustentacular-like ciliated cells showed phospho-α-syn deposits in the cytoplasm, more concentrated in the apical portion (Fig. 3c, d). Conversely, OM preparations from 12 non-PD patients or 12 normal controls showed only a faint positivity to both α-syn and phospho-α-syn (Fig. 3e, f).

### Diagnostic accuracy of CSF RT-QuIC

**Fig. 4:**
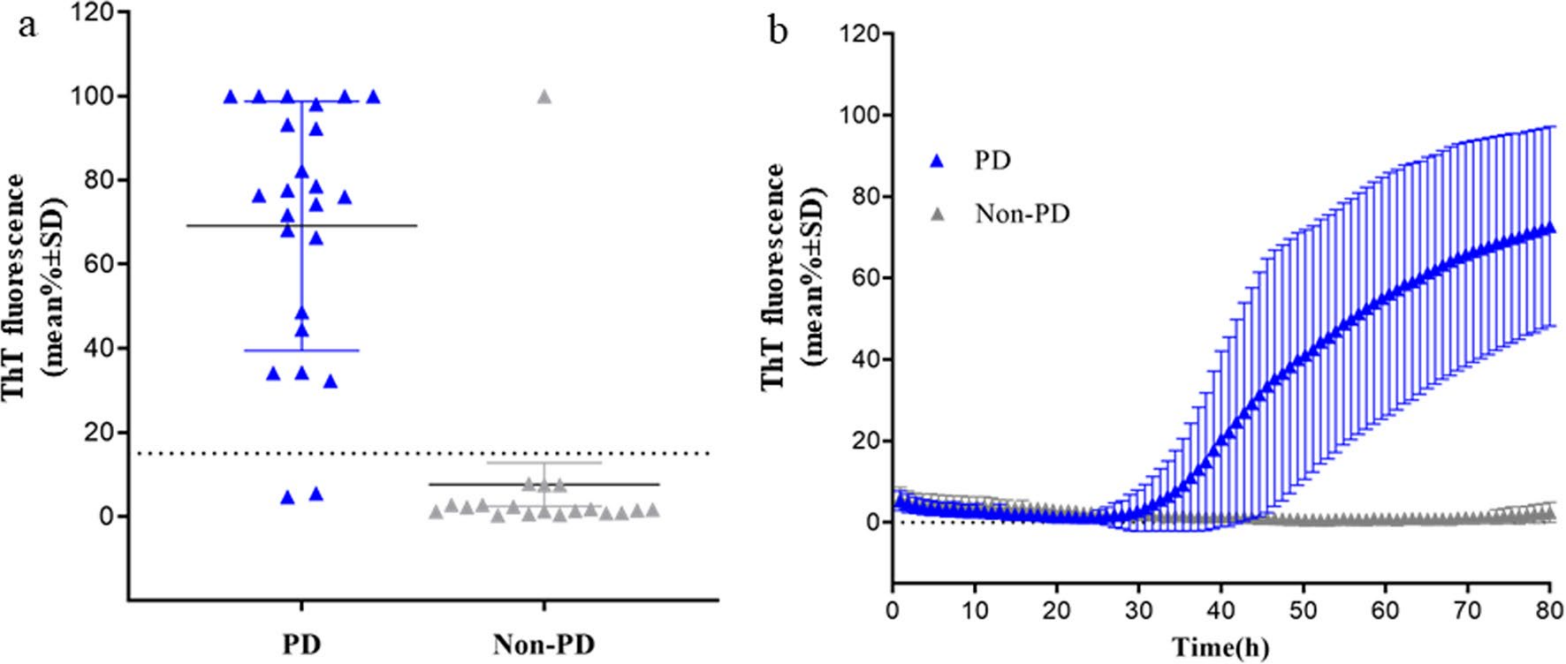
RT-QuIC detection of α-syn seeding activity in CSF samples of patients with PD and non-PD. **a** The average of relative ThT fluorescence from four replicate readings obtained from CSF of each individual cases with PD (*n* = 24) and non-PD (*n* = 19) at 80 h. Bars show the average ± SD for each group. The dashed line shows the fluorescence threshold for a positive result. **b** Traces represent the average percentage of ThT fluorescence readings from positive 22/24 CSF samples from patients with PD and 1/19 non-PD . The means (thick lines) with SD (thin lines) of those averages are shown as a function of RT-QuIC reaction time

Twenty-four patients with PD enrolled at the first round also underwent lumbar puncture and 22 CSF samples were positive for α-syn RT-QuIC (92%) (Fig. 4a, b). Nineteen CSF samples from patients with a clinical diagnosis of non-PD (5 with AD, 3 with PSP and 11 with CJD) were included as controls, of which one CSF sample from a patient with AD was positive for α-syn RT-QuIC (Fig. 4a, b).

RT-QuIC analysis of CSF provided a slightly higher diagnostic accuracy compared to OM (94% *vs* 82%). In 43 patients with both CSF and OM samples (31 collected from AN and 12 from MT), the RT-QuIC results did not differ (*P* = 0.9) between the two samples (*k* = 0.72, 95%CI 0.70–0.74). It should be noted that of the two CSF-negative samples, one was positive in paired OM while the other was negative in OM.

## Discussion

NS was originally targeted as an innovative procedure for intravital diagnosis of human prion disorders [28, 29]. More recently, α-syn RT-QuIC has been explored in NS of patients with DLB, PD and iRBD, providing variable diagnostic accuracies [11, 12]. However, a yet unexplored issue is the performance of the RT-QuIC assay in samples obtained from different OM areas, as done for other peripheral tissues during optimization of the sampling procedure. In patients with PD, OM samples had been always collected at the level of MT and RT-QuIC sensitivity was 46% in one study which included 63 patients, or 56% in another on 18 PD patients [10, 11].

Here, we obtained an overall agreement of 74% between RT-QuIC positivity and clinical diagnosis (32/43), higher than that obtained in previous studies. However, when analyzed separately, the RT-QuIC assay in NS^AN^ and NS^MT^ had sensitivity of 84% and 45%, respectively. The findings were reproduced in a second round when 23 additional PD patients underwent the RT-QuIC assay in both NS^AN^ and NS^MT^, providing a sensitivity of 78% and 43%, respectively. In conclusion, this is the first study showing that NS performed in two distinct areas of OM provided a significant difference in RT-QuIC sensitivity (*P* = 0.021) and that the higher RT-QuIC positivity was correlated to the higher distribution of β-III tubulin-positive olfactory cells in NS^AN^ [20, 21].

Notably, in patients with Parkinson’s disease dementia (PDD) (data not shown), DLB or prodromal DLB, the RT-QuIC sensitivity was ~ 80% regardless of the NS procedure, and no significant difference in the sensitivity between OM samples collected from AN and MT was observed [12]. Thus, it is tempting to hypothesize that in the presence of cognitive impairment, α-syn aggregates are involved in a larger area of OM.

Previous post-mortem studies have shown Lewy body (LB) pathology in the olfactory bulb but less frequently in the olfactory neuroepithelium of PD cases. In addition, α-syn aggregates or phospho-α-syn deposits were not detected in OM biopsy from subjects with PD. The lack of LB pathology was explained by the rapid turnover of olfactory cells, estimated to be few months, not sufficient for harbouring LB pathology [30, 31]. Here, we showed that α-syn and phospho-α-syn deposits, detected in the OM of PD patients but not in non-PD patients or controls, have seeding activity, indicating that even if LB pathology is not detected, α-syn is pathologically folded. It remains to be clarified whether in patients with PD, α-syn is highly expressed or has impaired degradation, and which metabolic dysfunctions lead to abnormal α-syn phosphorylation. It is well known that these aberrant changes of α-syn represent predisposing conditions for generating pathologic α-syn aggregates [32]. However, the phospho-α-syn deposition detected in the OM of PD patients should be further analyzed in a larger number of individuals.

Likely, the detection of α-syn aggregates in AN, a distinct area of OM, indicates that AN is a preferential site for α-syn aggregate formation. This occurrence might have a possible mechanical explanation. Normally, odorant molecules slow their flow in the superior area of the nasal cavity to allow their capture by olfactory receptors. This favors the exposure of AN olfactory receptor cells to environmental toxicants, which exerts cell stress damage contributing to α-syn misfolding [33]. A previous study showed that the olfactory bulb of patients with PD is affected by LB pathology in a gradient manner of progression, with neuronal loss at the level of glomeruli first, and subsequently in the anterior olfactory nucleus [34]. In mammals, the olfactory neurons are compartmentalized by expression of receptors in defined areas of the neuro-epithelium which projects to distinct regions of the olfactory bulb, preserving the spatial topography of the nasal epithelium in the glomerular sheet of the olfactory bulb [35–37]. Although this specific organization has not been clearly shown in humans, the preferential distribution of α-syn aggregates in the superior/anterior area of OM might find a correlation to LB pathology in the olfactory bulb of PD.

### Diagnostic accuracy of RT-QuIC in CSF and in the combination of CSF and OM

Here, we showed that the sensitivity of RT-QuIC in CSF was 94%, higher than that in the NS. The concordance of CSF RT-QuIC results with clinical diagnosis is comparable to that reported in previous studies, which showed a sensitivity ranging from 84% to 96% and specificity from 82% to 100% [5, 6, 38–42]. Notably, in this study, one of the two PD patients with negative results of CSF RT-QuIC, showed a positive result in OM (sampled from the AN). These findings support the assumption that a double tissue testing should be the ideal diagnostic approach. We previously showed in human prion disorders that the sensitivity of RT-QuIC in CSF and OM increased from 95% when the two samples were tested separately to 100% when both samples were tested [28, 29]. In patients with DLB, α-syn RT-QuIC assay in CSF and OM showed a concordance with clinical diagnosis of 94% and 86%, respectively, which increased to 100% when the results were combined. More recently, α-syn RT-QuIC in skin biopsies has also been considered supportive for CSF analysis [14]. However, compared to α-syn RT-QuIC testing in the skin, a greater diagnostic accuracy has been obtained by immunofluorescence detection of phospho-syn deposits in skin nerves of PD patients. These findings provide a further rationale for the use of NS in the diagnostic assessment of PD and related disorders [43].

This study is not without limitations. This is a clinical study and the results obtained need to be further confirmed in definite PD. Further, 20% of the PD patients were RT-QuIC negative in NS and 73% of these PD patients did not complain hyposmia. It might be not excluded that these PD patients represent a distinct phenotypic subgroup with relative sparing of OM or may be affected by other forms of Parkinsonisms [7]. These patients, as well as non-PD patients with NS positive for α-syn RT-QuIC, will be followed-up.

In conclusion, following NS optimization, the sensitivity of RT-QuIC in OM was increased to ~80%, comparable to that of skin biopsies. Specific protocols of sampling have been reported for skin biopsy, providing a sensitivity of 82.4% or 76.9% based on the number of skin samples obtained from each patient [17]. Further, NS allows collection of optimal OM samples with a single procedure, and can be done for multiple times, providing the optimal approach for follow-up studies.

## Conclusion

In this study**,** we optimized the protocol of NS in patients with PD and we showed that the sensitivity of α-syn RT-QuIC was significantly increased when OM was collected at the level of AN as compared to MT (~ 80% *vs* ~ 45%). However, the above sampling protocol did not provide significant differences in sensitivity of the test, when performed in patients with PDD or DLB. Taken together, these results suggest that, in PD, deposition of aberrant α-syn preferentially occurs in AN, which eventually spreads to the entire OM. In line with other studies, RT-QuIC analysis of CSF showed a sensitivity of 94%. Combining the CSF results with the results of NS can increase the diagnostic accuracy to nearly 100%.

## Supplementary Information

**Additional file 1: Fig. S1** Olfactory mucosa (OM) sample without substrate (recombinant α-syn and reaction buffer) (red trace) and substrate without OM (black trace) were tested by RT-QuIC.

## Data Availability

All the data generated or analysed during this study are included in this published article.

## Abbreviations

AD: Alzheimer disease
α-syn: α-Synuclein
CBS: Corticobasal syndrome
DLB: Dementia with Lewy bodies
MSA: Multiple system atrophy
OM: Olfactory mucosa
p-α-syn: Phosphorylated α-synuclein
PSP: Progressive supranuclear palsy
REM: Rapid eye movement
RT-QuIC: Realtime quaking-induced conversion
ThT: Thioflavin T
