# Correction: Olfactory swab sampling optimization for α-synuclein aggregate detection in patients with Parkinson’s disease

**DOI:** 10.1186/s40035-022-00312-2

**Published:** 2022-08-12

**Authors:** Matilde Bongianni, Mauro Catalan, Daniela Perra, Elena Fontana, Francesco Janes, Claudio Bertolotti, Luca Sacchetto, Stefano Capaldi, Matteo Tagliapietra, Paola Polverino, Valentina Tommasini, Giulia Bellavita, Elham Ataie Kachoie, Roberto Baruca, Andrea Bernardini, Mariarosaria Valente, Michele Fiorini, Erika Bronzato, Stefano Tamburin, Laura Bertolasi, Lorenzo Brozzetti, Maria Paola Cecchini, Gianluigi Gigli, Salvatore Monaco, Paolo Manganotti, Gianluigi Zanusso

**Affiliations:** 1 Department of Neurosciences, Biomedicine, and Movement Sciences, Policlinico G. B. Rossi, University of Verona, 37134 Verona, Italy; 2 Neurology Unit, Department of Medicine, Surgery and Health Sciences, Ospedale Cattinara, University of Trieste, 34128 Trieste, Italy; 3 Neurology Unit, University of Udine Academic Hospital, 33100 Udine, Italy; 4 Department of Surgical Sciences, Dentistry, Gynecology and Pediatrics, University of Verona, 37134 Verona, Italy; 5 Biocrystallography Laboratory, Department of Biotechnology, University of Verona, 37134 Verona, Italy; 6 Otolaryngology Unit, Department of Medicine, Surgery and Health Sciences, Ospedale Cattinara, University of Trieste, 34128 Trieste, Italy

## Correction: Translational Neurodegeneration (2022) 11:37 https://doi.org/10.1186/s40035-022-00311-3

In the original publication of this article [1], “AN” is missing in the column heading “Patients underwent NS at the (n = 46)” in Table 1 due to a typesetting error. The correct column heading should be “Patients underwent NS at the AN (n = 46)”. Moreover, the asterisk symbol * in “27 (84)*” and “5 (45)*” should be removed.

In addition, the phrase “using FLOQBrushes (Copan Italia, Brescia, Italy)” needs be added in the first sentence under the header “OM sample collection” of the Methods section.

The first sentence should be changed to:

NS was performed by otolaryngologists in each unit, independently, using FLOQBrushes (Copan Italia, Brescia, Italy).

The original article [1] was updated.
