# Supplementary material for: Olfactory swab sampling optimization for α-synuclein aggregate detection in patients with Parkinson’s disease
Source: Transl Neurodegener. 2022 Jul 28;11:37. doi: 10.1186/s40035-022-00311-3 (PMC9330656; doi:10.1186/s40035-022-00311-3)
Supplement: Supplementary file 1 — Additional file 1: Fig. S1 Olfactory mucosa (OM) sample without substrate (recombinant α-syn and reaction buffer) (red trace) and substrate without OM (black trace) were tested by RT-QuIC. [file 40035_2022_311_MOESM1_ESM.docx]

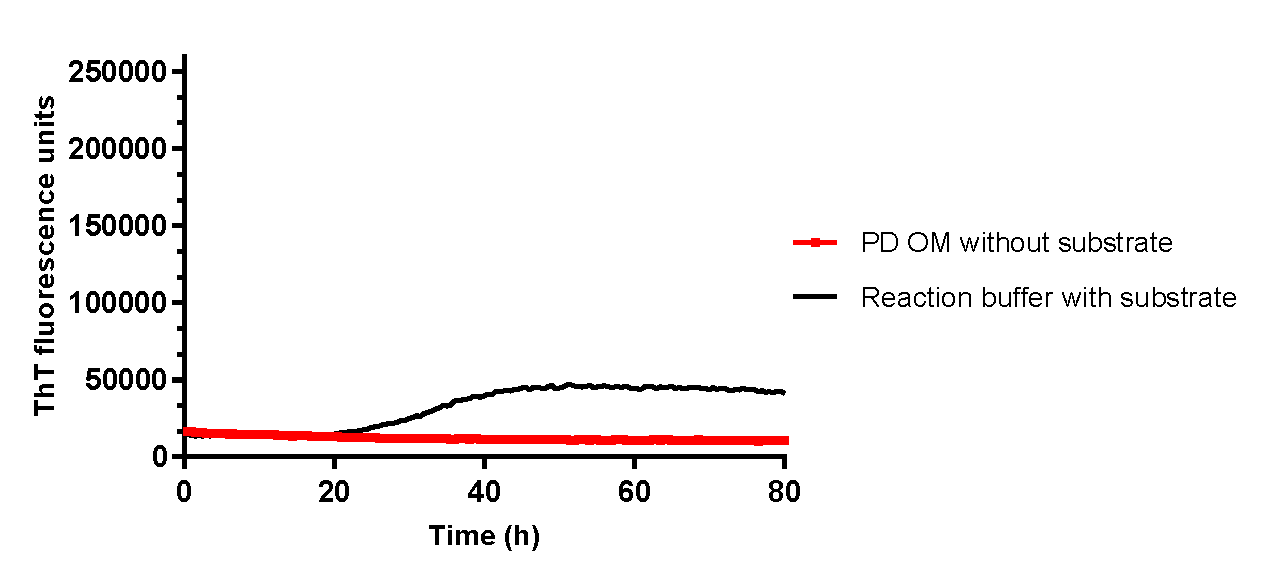


**Fig. S1** Olfactory mucosa (OM) sample without substrate (recombinant α-syn and reaction buffer) (red trace) and substrate without OM (black trace) were tested by RT-QuIC. Traces represent the relative average fluorescence readings.
